# The Role of Pharmacogenetics in the Therapeutic Response to Thiopurines in the Treatment of Inflammatory Bowel Disease: A Systematic Review

**DOI:** 10.3390/jcm12216742

**Published:** 2023-10-25

**Authors:** Aline C. Ribeiro, Pâmela S. A. S. Gerheim, Julio Maria Fonseca Chebli, Jorge Willian L. Nascimento, Priscila de Faria Pinto

**Affiliations:** 1 Department of Pharmaceutical Sciences, Faculty of Pharmacy, Federal University of Juiz de Fora, Juiz de Fora 36036-900, Minas Gerais, Brazil; alinecorrearibeiro@yahoo.com.br; 2 Department of Pharmacology, Federal University of Juiz de Fora, Juiz de Fora 36036-900, Minas Gerais, Brazil; pamela.souza@ufjf.br; 3 Division of Gastroenterology, Department of Internal Medicine, Federal University of Juiz de Fora, Juiz de Fora 36036-900, Minas Gerais, Brazil; julio.chebli@medicina.ufjf.br; 4 Laboratory of Clinical and Experimental Pharmacology, Department of Pharmacology, Federal University of Juiz de Fora, Juiz de Fora 36036-900, Minas Gerais, Brazil; 5 Department of Biochemistry, Institute of Biological Sciences, Federal University of Juiz de Fora, Juiz de Fora 36036-900, Minas Gerais, Brazil

**Keywords:** inflammatory bowel diseases, azathioprine, 6-mercaptopurine, pharmacogenetics, thiopurine S-methyl transferase

## Abstract

This study focuses on the use of thiopurines for treating inflammatory bowel diseases (IBD). These drugs undergo enzymatic changes within the body, resulting in active and inactive metabolites that influence their therapeutic effects. The research examines the role of genetic polymorphisms in the enzyme thiopurine S-methyltransferase (TPMT) in predicting the therapeutic response and adverse effects of thiopurine treatment. The TPMT genotype variations impact the individual responses to thiopurines. Patients with reduced TPMT activity are more susceptible to adverse reactions (AEs), such leukopenia, hepatotoxicity, pancreatitis, and nausea, which are common adverse effects of thiopurine therapy. The therapeutic monitoring of the metabolites 6-thioguanine nucleotides (6-TGN) and 6-methyl mercaptopurine (6-MMP) is proposed to optimize treatment and minimize AEs. Patients with higher 6-TGN levels tend to have better clinical responses, while elevated 6-MMP levels are linked to hepatotoxicity. Genotyping for TPMT before or during treatment initiation is suggested to tailor dosing strategies and enhance treatment efficacy while reducing the risk of myelosuppression. In conclusion, this study highlights the importance of considering genetic variations and metabolite levels in optimizing thiopurine therapy for IBD patients, focusing on balance therapeutic efficacy with the prevention of adverse effects and contributing to personalized treatment and better patient outcomes.

## 1. Introduction

Thiopurines, specifically azathioprine (AZA) and 6-mercaptopurine (6-MP) are, respectively, a prodrug and drug frequently used in the treatment of inflammatory bowel diseases (IBD). Such medicines undergo enzymatic modifications intracellularly, leading to the formation of active metabolites, 6-thioguanine nucleotides (6-TGN), and a major inactive metabolite, 6-methyl mercaptopurine (6-MMP), which is related to hepatotoxicity [1,2].

Thiopurines are effective for maintaining remission and as a steroid-sparing agent in the medium- and long-term in IBD, particularly when 6-TGN levels remain above 235 pmol/8 × 10^8^ RBC (red blood cells). However, the non-adherence rate of patients to therapy is around 17%, as shown by the undetectable or low intraerythrocytic levels of the metabolites [3,4,5,6].

Despite the beneficial effects of thiopurines on treating IBD, therapy is often limited by common adverse effects (AEs) inherent to the drugs or their metabolites. Around 10% to 30% of patients cannot tolerate therapy due to AEs such as myelotoxicity, hepatotoxicity, pancreatitis, and nausea. Some authors suggest that the by-product of the conversion of AZA to 6-MP, a nitroimidazole derivative, may be the primary cause of some of these AEs [3,7,8,9,10].

Thiopurine S-methyltransferase (TPMT) is a crucial enzyme involved in the metabolism of thiopurines; it catalyzes the S-methylation of the aromatic and heterocyclic sulfhydryl groups. Although the mechanism of action of thiopurines is not yet entirely clear, it is proposed that it can result from the incorporation of the 6-TGN metabolite into the cell’s DNA, impairing DNA synthesis and leading to cell apoptosis [1,2]. The gene encoding TPMT exhibits genetic heterogeneity, resulting in vast interindividual differences when considering clinical efficacy and toxicity profiles during treatment with thiopurines [1,11]. Several studies have pointed out that patients with polymorphisms in the TPMT gene related to decreased enzymatic activity are at greater risk of thiopurine-induced leukopenia [2,9].

These changes in TPMT activity have a significant population distribution profile. Approximately 0.3% of individuals in the general population have TPMT with low activity, while 10% have moderate activity, which leads to higher concentration of 6-TGN and an increased risk of severe myelosuppression or other toxic effects related to thiopurine treatment [3,6,8,12,13].

So far, three wild-type alleles and 39 variant alleles of this gene have been identified, most of which are associated with the decreased enzymatic activity of TPMT. Four genotypes, TPMT * 2, * 3A, * 3B, and * 3C, have been intensively investigated because they represent more than 95% of mutations and are associated with various degrees of reduction in enzyme activity. The TPMT * 3A is a haplotype that contains two non-synonymous SNPs: * 3B and * 3C [11], and the TPMT * 3A genotype is the most prevalent genotype in Caucasians (3.2–5.7%) and White Americans, followed by the TPMT * 3C genotype (0.5–1.5%). This genotype is also the most common variant in Africa, Ghana, and Asia, and the main variant type in Japan, with enzyme activity reduced by approximately 25% compared to the non-polymorphic allele [9,11,14,15,16,17,18,19]. The TPMT * 2 genotype, with a prevalence of 0.2–0.5%, is significantly more associated with AZA-induced leukopenia, followed by an unusual prevalence of TPMT * 3B, found in Chinese, Ghanaian, and Japanese populations. The TPMT * 3D and TPMT * 4 are found in a northern European family. The TPMT * 5 and TPMT * 6 are expressed in Koreans, the TPMT * 7 is found in European Caucasians, and the TPMT * 8 is found in African Americans [9,11,14,15,16,17,18,19].

Therefore, this study aims to conduct a systematic review to analyze the influence of the genetic polymorphisms of the TPMT enzyme on the therapeutic response related to thiopurine (azathioprine or mercaptopurine) in patients with IBD.

## 2. Materials and Methods

The systematic review was conducted according to the eligibility criteria (Table 1) and described following the Preferred Reporting Items for Systematic Reviews and Meta-Analyses (PRISMA 2020) [20,21]. The protocol is registered in the International prospective register of systematic reviews PROSPERO (# CRD42023397307).

### 2.1. Research Strategies and Sources of Information

Medical literature research was performed using MEDLINE, Scielo, Scopus, BVS Lilacs, and the Cochrane Library. The analysis included English, Portuguese, and Spanish articles published until October 2022 without ethnic restriction. Only original studies published in article format with an approach to the influence of genetic polymorphisms of the TPMT enzyme on the response to thiopurine therapy in patients with IBD were included.

We considered controlled and randomized clinical trials and retrospective or prospective multicenter studies using thiopurines to treat patients with IBD. We addressed the genetic variations related to the TPMT enzyme that metabolizes the drugs of interest.

Animal-related studies, in vitro studies, or studies unrelated to pharmacogenetics using thiopurines, case reports, and articles containing incomplete information, as judged by the reviewers, were excluded from this research. The research strategy used the following keywords: polymorphism genetic, pharmacogenetic, drug effects, azathioprine, thiopurine, and inflammatory bowel diseases.

### 2.2. Selection Process

Three researchers independently reviewed all articles by screening their titles and abstracts to assess their relevance. A second confirmatory screening was performed using the Rayyan App (http://rayyan.qcri.or, accessed on 3 September 2023).

Search terms were constructed based on PICOT. Terms related to Crohn’s disease, ulcerative colitis, inflammatory bowel diseases, azathioprine, 6-mercaptopurine or thiopurine, 6-methyl mercaptopurine, 6-thioguanine, genetic polymorphism, allelic variants, pharmacokinetic analysis, pharmacogenetics, genetic mutations, genotype, drug effects, therapy, immunosuppressive agents, adverse drug reactions, and metabolite monitoring were applied using free text and appropriate controlled vocabulary. Furthermore, search terms were combined within the same domain using ‘OR’ and across domains using ‘AND’.

### 2.3. Assessment of Risk of Bias

Two independent reviewers assessed the risk of bias in the included studies. The studies were classified as “low risk” and “concerning” (22 articles that did not make clear for how long the follow-up of patients occurred or had subjects that were lost during the studies, probably not introducing bias in the study), “moderate risk with some concerns” (14 articles with a short follow-up time of patients or had a follow-up rate of around 70–80% of patients recruited), and “high risk” (4 articles that were not blinded to interventions or had subjects lost for follow-up, likely introducing bias). This assessment was conducted for both the study and primary outcomes.

## 3. Results

### 3.1. Selection and Characteristics of the Studies

**Figure 1:**
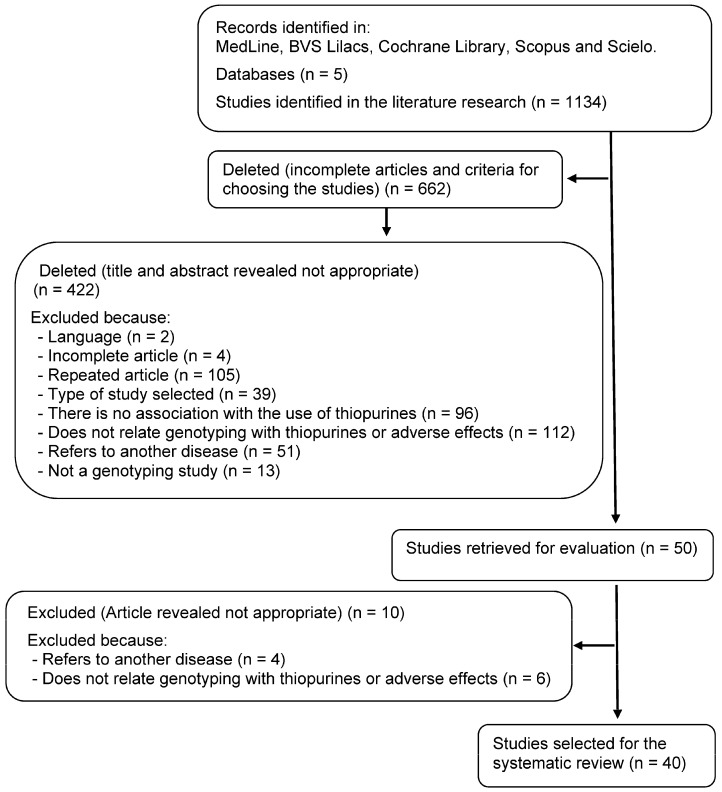
Flowchart of the article selection process.

The broad search strategy identified 1134 studies involving genetic polymorphisms related to drug therapy with thiopurines in patients with IBD. From the application of the inclusion and exclusion criteria previously defined, 662 articles were initially excluded (Figure 1). A total of 472 papers were evaluated in detail, and of these, 432 were excluded for different reasons, leaving 40 articles eligible for inclusion.

Figure 1 shows the flowchart used to select the analyzed articles, and Table 2 summarizes the detailed characteristics of the studies included in this systematic review.

The most included were cohort studies (38 studies, 95%), followed by randomized controlled trials (two studies, 5%). Most of the studies were conducted in Europe (England (three), Netherlands (three), Italy (three), Greece (two), Slovakia (two), Germany (one), and Spain (one)) and Asia (Japan (six), Korea (four), and China (five)), followed by the United States (three), and Oceania (New Zealand (two) and Australia (one)).

### 3.2. Effect of Genotype on the Profile of AE to Thiopurines

The analysis of the studies in the articles showed a high rate of patients (10 to 39%) with some AE, especially in the first three months of therapy. The most commonly reported AEs included dizziness, malaise, fever, pancreatitis, leukopenia, hepatotoxicity, gastrointestinal effects such as nausea, vomiting, abdominal discomfort, or reduced appetite, and a high occurrence of gastric intolerance. Less commonly reported adverse effects included dermatological issues such as skin reactions, hair loss, and warts, as well as myalgia, arthritis, susceptibility to infections, a potential risk of malignancy, and flu-like syndrome [1,2,6,9,20,23,27]. The occurrences of AEs in the participants of the selected studies can be found in Table 3.

An increased risk of thiopurine toxicity has been observed in patients with a TPMT deficiency in various conditions beyond IBD, such as hematologic malignancies, transplantation, and autoimmune diseases. Likewise, genetic variation in the activity of different enzymes involved in the thiopurine metabolism may explain part of the toxicity, which is not accounted for by variation in the TPMT activity [1,13,23,27,39]. The association between the presence or absence of the TPMT polymorphism and the AEs observed in the studies is presented in Table 4 and Table 5.

### 3.3. Effect of Genotype on Thiopurine Efficacy

Studies indicate that males are six times more likely to experience a reduced response to AZA compared to females. However, females have more AEs (52.6%), especially patients over 40. Additionally, females are two to three times more likely to develop leukopenia than younger females and males. Smokers are more susceptible to developing leukopenia and respond poorly to therapy [16,22,36,42]. The correlation between the expected clinical response and the reduced response with the presence or absence of the TPMT polymorphism in the participants of the selected studies is shown in Table 6.

## 4. Discussion

### 4.1. TPMT Genotypes and Adverse Effects

Our review provides a comprehensive analysis of TPMT genotypes and their association with adverse effects (AEs), unlike most previous work focused on the hematological toxicity of thiopurines.

Most dosing guidelines for AZA in the treatment of inflammatory bowel disease (IBD) are primarily based on studies involving Caucasian patients, where a daily dose of 2 to 3 mg/kg of AZA (Clinical Pharmacogenetics Implementation Consortium) or 1.5 to 2.5 mg/kg (European Crohn’s and Colitis Organization) is recommended for patients with regular TPMT activity. However, applying these guidelines to other ethnicities requires further evaluation in terms of therapeutic efficacy and toxicity. Japanese populations, for example, have shown that lower doses of AZA are sufficient to achieve clinical efficacy and therapeutic concentrations of 6-TGN [34,36]. In a study by Kim et al. (2010), it was observed that only 35.3% of patients could be treated with more than 2.0 mg/kg of AZA. Additionally, Coenen et al. (2015) reported a 40.0% discontinuation rate of thiopurine therapy due to AEs, which is relatively high when compared to other studies [6,32].

Although AZA is cost-effective, one of the most common and potentially severe AEs is myelosuppression, occurring in 3 to 7% of patients, leading to severe and possibly fatal infections [9,19]. It has been shown that leukopenia is the most common form of thiopurine-induced myelosuppression, with severe cases often emerging within the first month of therapy, but capable of occurring at any point during treatment [2,22,34,39,40,41]. Approximately 37% of the patients presented leukopenia with less than three months of treatment, 22.4% of the patients within 3 to 6 months, 13.8% within 6 to 12 months, 22.4% at 12 to 24 months, and 14.7% after 24 months of treatment with AZA/6-MP [9,18,32]. Furthermore, leukopenia can develop abruptly without any symptoms or warning signs, and its incidence can be as high as 47% in patients on thiopurine and 5-aminosalicylate (5-ASA) combination therapy compared to 16% in patients on thiopurine monotherapy [2,8,22,34].

Despite limited research, some studies have explored factors that can affect the activity of TPMT, showing that the induction of the enzyme with the use of thiopurines in up to 35% of patients and its inhibition by the use of some drugs such as acetylsalicylic acid, furosemide, and 5-ASA, mainly mesalazine and sulfasalazine, associated with thiopurines, may increase occurrences of AEs in up to 92% of associations. Salicylic acid-based drugs are thought to induce adverse reactions by reducing TPMT activity, raising the blood level of 6-TGN as the dose increases during the co-administration of 5-ASA [7,10,12,16,18,29,31,35].

Kim et al. (2010) pointed out that the high occurrence of bone marrow suppression during AZA/6-MP treatment could not be solely attributed to genotyping or TPMT activity. This suggests that myelosuppression is a multifactorial outcome, requiring further investigation [32,41]. Several factors, including the influence of the therapeutic dose of AZA and the co-administration of 5-ASA, which is commonly used to induce and maintain remission, suggest that 5-ASA inhibits TPMT and interacts with AZA, potentially leading to increased 6-TGN levels in 82% to 100% of patients, suggesting greater therapeutic efficacy, but also a higher risk of leukopenia [5,16,32,34,41].

In contrast to the decrease in 6-MMP levels, a statistically insignificant increase in the levels is observed among patients undergoing 5-ASA therapy. Some studies are not consistent with the hypothesis that this increase in 6-TGN and 6-MMP is due to TPMT inhibition, suggesting that 5-ASA may affect AZA and 6-MP metabolism through mechanisms not related to enzyme inhibition. Such inhibition of TPMT by 5-ASA medications and their metabolites occurs in vitro but not in vivo, as the inhibitor is removed during the washout steps of the assay [29,33,34,38]. Salicylic acid preparations are standard medications for IBD, and many patients with refractory IBD, requiring thiopurines, have likely already undergone treatment with 5-ASA. Thus, the dose of 5-ASA is unlikely to increase thiopurine-induced AEs significantly. However, patients with reduced TPMT activity should have this association evaluated cautiously [18].

Considering these findings, a likely interaction between 5-ASA and TPMT/6-TGN needs to be considered seriously, as patients with IBD, especially with active ulcerative colitis (UC), may be receiving high doses of 5-ASA, up to 4 g/day, and, with such a high dose, the inhibition of TPMT and increase in the mean 6-TGN levels cannot be disregarded, even with dose-adjusting thiopurines [12,29,38].

**Figure 2:**
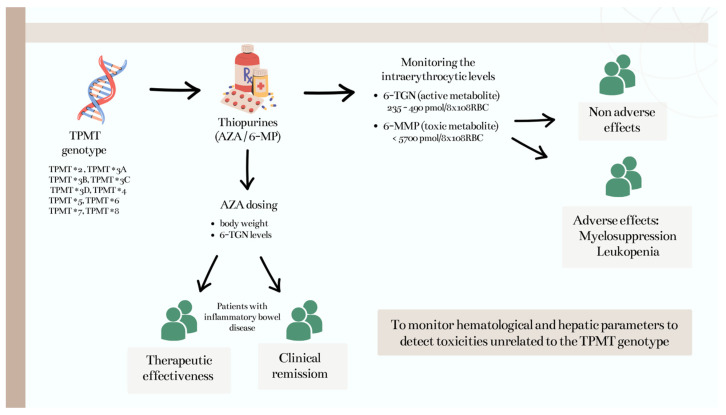
TPMT genotyping as a clinical tool for patients with DII receiving thiopurines.

Several studies that relate the reduced enzymatic activity of TPMT and the occurrence of severe AEs suggest that other factors could be involved in addition to the genetic variability of this enzyme [3,7,8,16,33,38]. Among the studies evaluated in this review, 21 (52%) directly correlated TPMT polymorphisms with the occurrence of adverse effects, particularly leukopenia, bone marrow toxicity, hepatotoxicity, pancreatitis, nausea, and vomiting (Figure 2). AEs such as fever, rash, myalgia, and arthralgia, thought to be allergic reactions, have been observed in patients able to tolerate AZA at therapeutic doses, leading researchers to question the nature of these events, suggesting that they are related to a type of hypersensitivity reaction, rather than TPMT pharmacogenetics [7].

### 4.2. Ethnic Variations in Thiopurine Dosing and Efficacy

The authors point out that differences between reports of the occurrence of AEs reported in studies can be explained by the racial backgrounds and the differing definitions of the threshold values adopted by the authors. For instance, many define the occurrence of these AEs when the leukocyte count is less than 3000/μL, neutrophils < 1500/μL, and lymphocytes < 1000/μL; alanine aminotransferase or aspartate aminotransferase when enzymatic activity exceeds twice the upper limit of the reference range; and for pancreatitis, the occurrence of severe abdominal pain and the three-fold elevation of serum amylase and/or lipase [9,13,25,26,27,32,41]. Another important finding is that pancreatitis has been observed as an idiosyncratic reaction in Western populations (2.8–7.4%) [9,14].

Fangbin et al. (2012) emphasize that evaluating the TPMT genotype has been considered a promising area in identifying the metabolic profiles of patients with a higher risk of AEs. However, there are studies reporting patients of different ethnicities who had leukopenia and did not have the TPMT variant allele, suggesting that the TPMT polymorphism does not efficiently predict AZA-induced leukopenia. Furthermore, the genetic polymorphism of TPMT differs significantly between populations [14,16,17,19].

### 4.3. Myelosuppression and Environmental Factors

Gazouli et al. (2010) reported that, in addition to genotype, environmental factors are important in influencing TPMT activity and the intraindividual variability observed in patient responses receiving thiopurines [3]. Consequently, an analysis of the TPMT genotypes before starting treatment may be useful for predicting myelosuppression and other AEs in IBD patients with TPMT polymorphisms, in addition to measurements of the enzyme activity and concentration of 6-TGNs, to ensure the safe use of AZA/6-MP [3,8,13,16,24]. The research indicates that TPMT activity varies approximately four-fold between the normal and intermediate metabolizers and varies inversely with the 6-TGN concentrations, suggesting that TPMT activity explains only 30% of the variation in the thiopurine dose, suggesting the involvement of other factors. Additional factors such as other drug treatment, age of red blood cells, and transfusions also influence TPMT enzyme activity [7,10,12,16,29,31,35].

Thiopurine treatment presents challenges due to the need for a dose–response relationship and a lengthy delay before therapeutic efficacy becomes evident. As for a dose–response relationship, the establishment of this profile is complicated by the genetic polymorphism associated with the activity of the TPMT enzyme [12]. Overall, a complete clinical response to AZA occurs in around 38% of patients, with a notable connection between the lower neutrophil counts and the response to AZA [23]. Previous studies on the influence of TPMT have focused on predicting toxicity in those with a TPMT deficiency (heterozygotes and homozygotes for the TPMT variant) at a high risk of severe neutropenia and other AEs, even at standard doses of AZA. However, high TPMT activity may also predict a poor clinical response due to a preference for 6-MP methylation over 6-TGNs bioactivation [13,23].

Very high TPMT activity can be a reliable predictor of a need for allopurinol co-therapy, or an alternative immunosuppressant to be recommended to prevent severe myelotoxicity, as allopurinol potently inhibits xanthine oxidase (XO), necessitating 25–33% of the standard daily dose of AZA or 6-MP [3,16,17,18,23,34,40]. Especially in these situations, the therapeutic monitoring of the metabolites 6-TGN and 6-MMP is an option that can help optimize drug therapy and minimize AEs; however, it needs to be addressed. In contrast, patients diagnosed with IBD, experiencing disease exacerbation during thiopurine maintenance, are generally subjected to a change of therapy to biologics, with no further attempt to optimize thiopurine dosing based on metabolite levels [4,16,34].

### 4.4. TPMT Genotypes, Metabolite Levels, and Clinical Response

Therapeutic monitoring is the only method to reveal non-compliance with thiopurine therapy, playing an important role in the case of refractory IBD. Theoretically, zero or deficient levels of 6-TGN could result from other factors such as thiopurine malabsorption, unknown enzyme defects, or enzymes with extremely high activity in the thiopurine metabolic pathway [4,8,12]. In recent years, the concentrations of the thiopurine metabolites, 6-TGN and 6-MMP, associated with a clinical response have been described by several studies, defining a therapeutic range between 235 and 490 pmol/8 × 10^8^ RBC. Patients with 6-TGN levels exceeding 490 pmol/8 × 10^8^ RBC are at an increased risk of leukocytopenia, whereas higher 6-MMP levels above 5700 pmol/8 × 10^8^ RBC are associated with hepatotoxicity [4,5,29].

An analysis comparing patients with different 6-TGN values and the occurrence of a clinical response showed that there was a difference between the groups, where 74% of patients with 6-TGN > 100 pmol/8 × 10^8^ RBC showed a clinical response compared to 46% with 6- TGN < 100 pmol/8 × 10^8^ RBC, increasing the probability of success in the clinical response of patients by 4.6 times. These results were corroborated by other similar studies where patients with higher levels of 6-TGN had a successful clinical response (57.6%, 313 pmol/8 × 10^8^ RBC versus 42.4%, 209 pmol/8 × 10^8^ RBC) (85%, greater than 225 pmol/8 × 10^8^ RBC vs. 17%) [5,23,25,26,27]. When assessing the AEs in patients with high concentrations of 6-TGN (above 286 pmol/8 × 10^8^ RBC), 22.6 to 30.0% had leukopenia, corroborating the literature which states that the concentration of intraerythrocytic 6-TGN, not the thiopurine dose, is significantly and independently associated with the therapeutic response. Other factors were evaluated but unrelated, such as age, sex, and type of disease (CD or UC) [5,16,25,26,27,29].

If 6-TGN concentrations are used to indicate the likely therapeutic efficacy, the actual difference in dose may be three-fold instead of two-fold, relative to intermediate metabolite status subjects, as suggested by some investigators and guidelines; however, these may require half the dose of patients with normal metabolizers. Thus, 6-TGN concentrations, as a clinical point above 235 to 260 pmol/8 × 10^8^ RBC, are associated with a three-fold more significant likelihood of remission [28]. Although these studies did not find a correlation between the metabolite levels and thiopurine doses resulting from differences in an inter-individual metabolism, in clinical practice, metabolites are dosed on a dose per patient body weight basis (AZA 2.0–3.0 mg/kg and 6-MP 1.0–1.5 mg/kg). However, patients with leukopenia may not reach the recommended AZA dose [4,12,32,36]. Chang et al. (2019) reported in their study that, although the duration of patient follow-up or cumulative drug dosage did not show a statistical difference according to TPMT genotyping, patients who underwent genotyping before starting treatment had a lower number of outpatient visits (7.8 ± 3.2 vs. 9.0 ± 3.9) and required a lower thiopurine dosage, discontinuation, or dose reductions (15.3% vs. 33.7%) during the study period [2].

In our review, some observed limitations included the characteristics of the published literature available for analysis. There is a high degree of heterogeneity among the included studies, which may be attributed to differences in the study design (clinical and randomized cohort studies), variations in the patient populations across the reported studies, and disparities in the definition of adverse effects. Further prospective multicenter studies are required to elucidate disease-specific variations in polymorphisms, especially within specific populations. Additionally, these studies can help identify new polymorphisms that could potentially explain leukopenia in patients lacking these recognized genetic variants.

Finally, we emphasize the importance of genotyping before or during the initial phase of treatment with thiopurines to ensure the continuity of treatment, monitor severe AEs, and contribute to reducing the medical budget and laboratory tests, which are usually necessary. Previous knowledge of TPMT genotyping is an important predictor of clinical response and may significantly reduce the occurrence of myelosuppression or thiopurine-induced leukopenia. However, genotyping before initiating thiopurine therapy cannot replace the current practice of periodically monitoring WBC and neutrophil counts [2,7,9,13,14,17,30].

## 5. Conclusions

Thiopurines such as AZA and 6-MP are effective in maintaining clinical remission in patients with IBD, but the long-term use of these drugs is associated with serious AEs, particularly bone marrow suppression.

Monitoring the intraerythrocytic levels of 6-TGN (active metabolite) and 6-MMP (toxic metabolite) in patients with IBD undergoing remission or maintenance therapy allows health professionals to be able to prevent or reduce the occurrence of AEs such as myelosuppression and other toxic effects. The quantification of AZA metabolites emerges as a valuable clinical tool for enhancing the precision and personalization of AZA therapy in IBD. While AZA dosing traditionally considers body weight, studies have revealed a limited correlation between dose per weight and 6-TGN levels. Therefore, it is advisable to routinely measure 6-TGN levels, even for patients adhering to the recommended AZA dosage. This practice provides valuable insights into the therapeutic effectiveness of the drug, allowing for more assertive treatment decisions and improved patient outcomes.

TPMT genotyping is also useful to suggest the most appropriate dose of thiopurines to start the treatment and prevent myelotoxicity and other AEs in patients with IBD, being essential to perform it at least in patients with IBD where AZA therapy is considered for treatment. However, healthcare professionals should also monitor patients treated with thiopurines for biochemical, hematological, and hepatic parameters to detect toxicities. 

## Figures and Tables

**Table 1:** Eligibility criteria of included studies.

Eligibility Criteria
Kind of study: clinical studies; controlled and randomized clinical trials; comparative studies; multicentric studies; observational and cross-sectional studies; genetic variations related to the enzyme that metabolizes the drugs of interest (AZA or 6-MP); TPMT enzyme genotype availability.
Search: MedLine; BVS Lilacs; Cochrane Library; Scopus; and Scielo; Main keywords (pharmacogenetic; polymorphism; thiopurine; inflammatory bowel diseases).
Population: patients diagnosed with Inflammatory Bowel Disease.
Intervention: on treatment with thiopurines for at least three months.
Language: originally in English.

**Table 2:** Characterization of the selected studies regarding the evaluated clinical outcome (adverse effects and clinical response).

Authors (Year)	Country	Sample (n)	DII CD/UC	Gender M/F	Age (Years)	Dose of AZA or 6-MP (Lowest–Highest Dose)	TPMT Alleles Evaluated	Metabolites Evaluated
AL-Judaibi et al. (2016) [22]	England	53	35/18	32/21	41.0 (19–80)	1.5 mg/Kg/day	* 1, * 3A	-
Ansari et al. (2008) [23]	England	207	117/90	92/115	40.3 (18–80)	2.0 (1.88–2.38) mg/Kg/day	* 1, * 3A, * 3B, * 3C	6-TGN
Ban et al. (2008) [24]	Japan	70	20/70	47/23	38.3 (17–79)	50 mg/day	* 1, * 3C	-
Bayoumy et al. (2021) [10]	Netherlands and UK	316	154/147/15 ^a^	121/195	45 (34–58)	20 (10–40) mg/day	* 1, * 2, * 3A	-
Chang et al. (2019) [2]	Japan	164	85/68	98/66	38.7 (20–80)	1.7 (1.1–2.3) mg/Kg/day	* 1, * 2, * 3B, * 3C	-
Coelho et al. (2016) [1]	England	78	67/23	56/44	<18 anos	1–5.2 mg/Kg/day 6-MP: 1–1.5 mg/Kg/day	* 1, * 3A, * 39	-
Coenen et al. (2015) [6]	Netherlands	783	356/253	354/429	41.0 ± 15.8	2.0 (0.5–3.01) mg/Kg/day	* 1, * 2, * 3A, * 3C	-
Coucoutsi et al. (2017) [11]	Greece	223	113/110	126/97	53.0 (16–90)	-	* 1, * 2, * 3A, * 3B, * 3C	-
Dubinsky et al. (2002) [25]	USA	51	35/16	30/21	36.6 (14–65)	2.5 (0.5–8.0) mg/Kg/day	-	6-TGN
Dubinsky et al. (2000) [26]	USA	92	79/13	41/51	11.5 (1–18)	6-MP: 1.3 (0.4–2.4) mg/Kg/day	* 1, * 3A, * 3B, * 3C	6-TGN
Fangbin et al. (2012) [14]	China	199	160/39	133/66	31.8 (18–80)	50 mg/day 6-MP: 25 mg/day	* 1, * 2, * 3A, * 3B, * 3C	-
Fangbin et al. (2016) [27]	China	132	102/30	76/56	34.0 (18–72)	50 mg/day 6-MP: 25 mg/day	* 1, * 2, * 3A, * 3B, * 3C	6-TGN
Gardiner et al. (2008) [28]	New Zealand	69	53/16	34/35	39.2 (35–43)	1.6 (1.46–1.81) mg/kg/day	* 1, * 3	6-TGN
Gazouli et al. (2010) [3]	Greece	97	69/17/11 ^a^	40/57	11.0 (3–16)	1.4 (0.3–2) mg/kg/day	* 1, * 2, * 3A, * 3B, * 3C	-
Gearry et al. (2003) [7]	New Zealand	56	39/17	27/29	40.6 (17–74)	-	* 1, * 2, * 3A, * 3C	-
Gilissen et al. (2011) [4]	Netherlands	100	57/40/3 ^a^	60/40	42.3 (15–79)	1.8 (0.6–3.0) mg/Kg/day6-MP: 0.8 (0.5–1.2)	-	6-TGN/6-MMP
Hanai et al. (2010) [12]	Japan	257	-/257	-	36.7 (14–68)	6-MP: 20–30 mg/day	-	6-TGN
Hande et al. (2006) [29]	USA	126	98/28	67/59	22 (6–79)	1.8 ± 0.6 mg/kg/day 6-MP: 1.2 ± 0.4 mg/kg/ day	-	6-TGN/6-MMP
Hibi et al. (2003) [30]	Japan	82/22	35/47	17/5	45.6 (28–64)	0.6–1.2 mg/Kg/day 6-MP: 50 mg/day	* 1, * 2, * 3A, * 3C	6-TGN
Hlavaty et al. (2013) [31]	Slovakia	220	19	119/101	37.1 ± 12.4	3.8 + 5.5 mg/Kg/day	* 1, * 2, * 3A, * 3B, * 3C	-
Kim et al. (2010) [32]	Korea	286	228/34/24 ^b^	187/99	25.7 ± 9.3	1.8 (0.56–3.26) mg/Kg/day6-MP: 1.5 (0.82–2.27)	* 1, * 2, * 3A, * 3B, * 3C	-
Lee et al. (2017) [5]	Korea	140	140	114/26	33.6 ± 8.8	1.2 ± 0.5 mg/Kg/day 6-MP: 0.6 ± 0.3 mg/Kg/day	* 1, * 3	6-TGN
Lee et al. (2015a) [33]	Korea	132	99/33	95/37	17 (15–18)	1.1 (0.81–1.30) mg/kg/day	* 1, * 3C, * 6, * 16	6-TGN/6-MMP
Lee et al. (2015b) [34]	Korea	137	103/34	98/39	17 (15–18)	1.0 (0.8–1.03) mg/kg/day	* 1, * 3C, * 6, * 16	6-TGN/6-MMP
Lindqvist et al. (2006) [35]	Sweden	54	-	-	-	2.5 mg/kg/day 6-MP: 1.25 mg/kg/day	* 1, * 3A	-
Odahara et al. (2015) [16]	Japan	48	19/29	29/19	34.2 ± 13.6	1.0 mg/kg/day	* 1,* 3A,* 3C,* 3D, * 4,* 5,* 6,* 7,* 8	6-TGN/6-MMP
Palmieri et al. (2007) [15]	Italy	422	250/172	227/195	39 (21–54)	1.6–3.2 mg/kg/day	* 1, * 3A, * 3B e * 3C	-
Ribaldone et al. (2019) [17]	Italy	200	120/80	116/84	33 (13–67)	0.5–1 mg/kg/day	* 1, * 2, * 3A, * 3B, * 3C	-
Sheffield et al. (2009) [36]	Australia	132	-	-	(18–80)	1.9 (1.6–2.0) mg/kg/day	-	6-TGN/6-MMP
Stocco et al. (2005) [8]	Italy	70	38/31/1 ^a^	34/36	14.2 (0.8–38.8)	2.0 (1.0–4.0) mg/kg/day	* 1, * 2, * 3A, * 3B, * 3C	-
Teml et al. (2005) [37]	Germany	20	-/14/6 ^a^	7/13	45 (19–75)	80 mg/day	* 1, * 2, * 3A, * 3B, * 3C	6-TGN/6-MMP
Uchiyama et al. (2009) [18]	Japan	16	8/8	9/7	39.1 ± 15.4	50 mg/day 6-MP 30 mg/day	* 1, * 2, * 3A, * 3B, * 3C, * 3D, * 4, * 5, * 6, * 7 e * 8	6-TGN/6-MMP
von Ahsen et al. (2005) [38]	Germany	71	71/-	31/40	36.0 ± 11.6	2.5 mg/kg/day	-	6-TGN/6-MMP
Wang et al. (2018) [19]	China	219	176/39/4 ^a^	160/59	33.4 ± 13.1	1.0–2.0 mg/kg/day	* 1, * 3C	-
Winter et al. (2007) [39]	UK	130	69/61	70/60	45	1.6 mg/kg/day	* 1, * 2, * 3A, * 3B, * 3C	-
Wroblova et al. (2012) [9]	Czech Republic and Slovakia	188	137/41/10 ^a^	107/81	37.3 (20–71)	1.4–2.0 mg/kg/day	* 1, * 2, * 3A, * 3B, * 3C	-
Zabala-Fernández et al. (2011) [13]	Spain	232	156/76	115/117	32.6 (8–70)	2.3 (1.5–3.0) mg/kg/day	* 1, * 2, * 3A, * 3B, * 3C	-
Zelinkova et al. (2006) [40]	Netherlands	262	195/67	103/159	39 (17–87)	132 (50–250) mg/day	* 1, * 2, * 3A, * 3B, * 3C	-
Zhu e Cao (2012) [41]	China	52	49/3	27/25	34 (16–77)	1.4 (0.9–2.2) mg/Kg/day	* 1, * 2, * 3A, * 3B, * 3C	-
Zhu et al. (2016) [42]	China	253	253/-	185/68	-	1.8 (0.5–3.1) mg/Kg/day	* 1, * 2, * 3A, * 3B, * 3C	-

Note: IBD: “^a^” undetermined colitis; “^b^” Intestinal Behcet’s disease; “-“: not described or not evaluated. Abbreviations: CD: Crohn’s disease; UC: Ulcerative colitis; M: Male; F: Female; AZA: Azathioprine; 6-MP: 6-Mercaptopurine; TPMT: Thiopurine S-methyl-transferase enzyme; 6-TGN: 6-Thioguanine nucleotides; 6-MMP: 6-methyl mercaptopurine.

**Table 3:** Occurrence of adverse effects in the participants of the selected studies.

Authors (Year)	Total Patients with EAs	Myelotoxicity	Hepatotoxicity	Leukopenia	Lymphopenia	Neutropenia	Thrombocytopenia	Hepatitis	Pancreatitis	Skin irritation/ Hair Loss	Arthralgia	Gastrointestinal Intolerance/Nausea/Vomiting	Flu-Like Symptoms	Discontinued/ Adjusted Dose
AL-Judaibi et al. (2016) [22]	17 (32.1%)	4/3 * (7.5%)										13 (24.6%)	2 (3.8%)	7 (13.2%)
Ansari et al. (2008) [23]	95 (44.2%)	7 (3.2%)	7 (3.2%)					8 (3.7%)		8 (3.7%)		76 (59.9%)	11 (5.1%)	83 (39.0%)
Ban et al. (2008) [24]				7 (10.0%)										
Bayoumy et al. (2021) [10]				28 (8.9%)			14 (4.4%)							
Chang et al. (2019) [2]		45/1 * (27.4%)		30 (16.5%)								42 (23.1%)		42 (23.1%)
Coelho et al. (2016) [1]	28 (21.9%)											1 (1.3%)		40 (40.0%)
Coenen et al. (2015) [6]	559 (71.4%)		203 (26.6%)	58 (7.5%)			3 (0.4%)	41 (5.2%)		171 (21.8%)	132 (16.9%)	448 (57.2%)		313.2 (40.0%)
Coucoutsi et al. (2017) [11]	23 (25.3%)					13 (58.3%)		5 (21.7%)		2 (8.7%)				23 (25.3%)
Dubinsky et al. (2002) [25]	19 (37.2%)		12 (24.0%)	8 (15.7%)										
Dubinsky et al. (2000) [26]	36 (39.1%)	1 (1.1%)	16 (17.4%)	13 (14.1%)										1 (1.1%)
Fangbin et al. (2012) [14]	50 (25.1%)		1 (0.5%)	36 (18.1%)		3 (1.5%)						6 (3.0%)	7 (3.5%)	
Fangbin et al. (2016) [27]	30 (22.7%)			26 (19.7%)								3 (2.3%)	1 (0.8%)	16 (12.1%)
Gardiner et al. (2008) [28]	22 (31.9%)		6 (8.7%)						2 (2.9%)			4 (5.8%)	8 (11.6%)	16 (23.2%)
Gazouli et al. (2010) [3]	10 (10.3%)	1 (1.0%)		6 (6.2%)					3 (3.1%)					13 (13.4%)
Gearry et al. (2003) [7]	56 (100%)	4 (7.1%)						18 (32.1%)	5 (8.9%)	13 (23.2%)		12 (21.4%)		56 (100%)
Hanai et al. (2010) [12]	18 (7.0%)	6 (2.3%)						5 (1.9%)	4 (1.6%)	3 (1.2%)				
Hibi et al. (2003) [30]	18 (12.8%)	12 (8.5%)		1 (0.7%)				1 (0.7%)		1 (0.7%)			2 (1.4%)	5 (3.5%)
Hlavaty et al. (2013) [31]	75 (34.1%)	32 (14.5%)	24 (10.9%)	26 (11.8%)					6 (2.7%)	2 (0.9%)		7 (3.2%)		43 (19.5%)
Kim et al. (2010) [32]				116 (40.6%)			2 (0.7%)						9 (3.1%)	8 (2.8%)
Lee et al. (2017) [5]				38 (27.1%)				1 (0.7%)		2 (1.4%)		13 (9.3%)		
Lee et al. (2015a) [33]				21 (15.9%)	40 (30.3%)	24 (18.2%)		4 (3.0%)	1 (0.8%)	16 (12.1%)	5 (3.8%)	15 (11.4%)		
Lee et al. (2015b) [34]				21 (15.3%)	40 (29.2%)	24 (17.5)		4 (3.0%)	1 (0.7%)	16 (11.7%)	5 (3.6%)	15 (10.9%)		
Lindqvist et al. (2006) [35]	39 (72.2%)	9 (16.7%)												27 (50.0%)
Odahara et al. (2015) [16]	14 (29.2%)			10 (20.8%)				1 (2.1%)		4 (8.3%)				12 (25.0%)
Palmieri et al. (2007) [15]	81 (19.2%)		12 (16.4%)	23 (31.5%)					16 (21.9%)	3 (4.1%)		9 (12.3%)	18 (24.7%)	
Ribaldone et al. (2019) [17]	60 (30.0%)			6 (10.0%)				24 (40.0%)	28 (46.7%)			2 (3.3%)		60 (30.0%)
Stocco et al. (2005) [8]	19 (27.1%)	7 (10.0%)	6 (8.6%)						4 (5.7%)		1 (1.4%)			19 (27.1%)
Teml et al. (2005) [37]				2 (10.0%)					1 (5.0%)	3 (15.0%)	4 (20.0%)	2 (10.0%)		8 (40.0%)
Uchiyama et al. (2009) [18]				12 (75.0%)				4 (25.0%)		5 (31.3%)		1 (6.3%)		
von Ahsen et al. (2005) [38]			3 (4.2%)						2 (2.8%)			8 (11.3%)		13 (18.3%)
Winter et al. (2007) [39]	44 (33.8%)	4 (3.1%)	9 (6.9%)	10 (7.7%)					1 (0.8%)	4 (3.1%)		14 (10.8%)	6 (4.6%)	33 (25.4%)
Wroblova et al. (2012) [9]	44 (23.4%)	34 (18.1%)	4 (2.1%)						2 (1.1%)			4 (2.1%)		
Zabala-Fernández et al. (2011) [13]	75 (32.3%)	15 (6.5%)	5 (2.2%)						19 (8.2%)	14 (6.0%)	6 (2.6%)	16 (6.9%)		72 (31.0%)
Zelinkova et al. (2006) [40]		12 (4.6%)	11 (4.2%)	7 (2.7%)			5 (1.9%)							21 (8.0%)
Zhu e Cao (2012) [41]		5 (9.6%)	1 (1.9%)	5 (9.6%)										6 (11.5%)
Zhu et al. (2016) [42]				65 (25.7%)		22 (8.7%)				6 (2.4%)				

Note: Myelotoxicity (Severe < 1500/mm^3^); Leukopenia (WBC < 3000/μL); Neutropenia (neutrophils < 1500/μL); Lymphopenia (lymphocytes < 1000/μL). Thrombocytopenia (PLT < 100,000/μL); Anemia (Hb < 10 g/dL); Pancreatitis (severe abdominal pain and 3-fold elevation of serum amylase and/or lipase); Hepatotoxicity (alanine aminotransferase or aspartate aminotransferase enzyme activity > 2 times the upper limit of the reference range); * Severe myelotoxicity.

**Table 4:** Association between TPMT polymorphism and types of adverse effects.

Authors (Year)	TPMT Genes		Myelotoxicity	Hepatotoxicity	Gastrointestinal Intolerance	Skin Irritation/Hair Loss	Pancreatitis	Leukopenia	Neutropenia	Nausea/Vomiting
AL-Judaibi et al. (2016) [22]	* 1/* 1	48 (90.6%)								
	* 1/* 3A	5 (9.4%)	2 (40.0%)						1 (20.0%)	
Ansari et al. (2008) [23]	* 1/* 1	200 (91.3%)	7 (8.4%)	8 (9.6%)	34 (40.1%)			7 (10.0%)		13 (7.0%)
	variants	19 (8.7%)	5 (26.3%)		8 (42.1%)	2 (10.5%)				7 (37.0%)
Ban et al. (2008) [24]	* 1/* 1	110 (99.1%)	7 (63.6%)						1 (0.9%)	
	* 1/* 3C	1 (0.9%)								
Coenen et al. (2015) [6]	* 1/* 1	705 (90.0%)								
	* 1/* 2	7 (0.9%)								
	* 1/* 3A	58 (7.4%)								
	* 1/* 3C	12 (1.5%)						1 (8.3%)		
	* 3A/* 3A	1 (0.1%)								
Coucoutsi et al. (2017) [11]	* 1/* 1	206 (92.4%)		5 (21.7%)					10 (43.5%)	
	* 1/* 2	4 (1.8%)								
	* 1/* 3A	6 (2.7%)							2 (8.7%)	
	* 1/* 3B	3 (1.3%)							1 (4.3%)	
	* 1/* 3C	4 (1.8%)								
Dubinsky et al. (2000) [26]	* 1/* 1	35 (97.2%)						12 (13.0%)		
	variants	1 (2.8%)						1 (1.1%)		
Fangbin et al. (2012) [14]	* 1/* 1	197 (99.0%)		1 (0.5%)				32 (16.1%)		6 (3.0%)
	* 1/* 3C	2 (1.0%)						4 (2.0%)		
Fangbin et al. (2016) [27]	* 1/* 1	130 (98.5%)								
	* 1/* 3C	2 (1.5%)						2 (1.5%)		
Gazouli et al. (2010) [3]	* 1/* 1	86 (88.7%)					3 (3.1%)	6 (6.2%)		
	* 1/* 2	3 (3.1%)								
	* 1/* 3A	2 (2.1%)								
	* 1/* 3B	4 (4.1%)								
Hlavaty et al. (2013) [31]	* 1/* 1	205 (93.2%)	62 (30.2%)	23 (11.2%)	7 (3.4%)		6 (2.9%)	18 (8.8%)		36 (17.6%)
	* 1/* 3A	13 (5.9%)	13 (86.7%)	1 (6.7%)	0 (0.0%)		0 (0.0%)	8 (53.3%)		1 (6.7%)
	* 1/* 3C	1 (0.5%)								
	* 3A/* 3A	1 (0.5%)								
Kim et al. (2010) [32]	* 1/* 1	279 (97.6%)						111 (39.8%)		
	* 1/* 3C	7 (2.4%)						5 (71.4%)		
Lee et al. (2017) [5]	* 1/* 1	135 (96.4%)						30 (21.4%)		
	* 1/* 3	5 (3.6%)						20 (14.3%)		
Ribaldone et al. (2019) [17]	* 1/* 1	192 (96.0%)		24 (40.0%)			26 (43.3%)	6 (10.0%)		2 (3.3%)
	* 1/* 2	2 (1.0%)								
	* 1/* 3A	6 (3.0%)					2 (3.3%)			
Stocco et al. (2005) [8]	* 1/* 1	65 (92.9%)	7 (10.0%)							
	* 1/* 2	1(1.4%)								
	* 1/* 3A	4 (5.7%)								
Uchiyama et al. (2009) [18]	* 1/* 1			4 (25.0%)		5 (31.3%)		12 (75.0%)		1 (6.3%)
von Ahsen et al. (2005) [38]	* 1/* 1	66 (93.0%)								
	* 1/* 2	1(1.4%)								1(1.4%)
	* 1/* 3A	4 (5.6%)								4 (5.6%)
Wang et al. (2018) [19]	* 1/* 1	216 (98.6%)							18 (22.8%)	
	* 1/* 3C	3 (1.4%)							1 (05%)	
Winter et al. (2007) [39]	* 1/* 1	119 (91.5%)		8 (6.2%)		3 (2.3%)		9 (6.9%)		14 (10.7%)
	* 1/* 3A	8 (6.2%)		1 (0.8%)		1 (0.8%)	1 (0.8%)			
	* 1/* 3C	3 (2.3%)						1 (0.8%)		
Wroblova et al. (2012) [9]	* 1/* 1	172 (91.5%)	26 (13.8%)	3 (1.6%)	3 (1.6%)		2 (1.1%)			
	* 1/* 3A	13 (6.9%)	8 (4.3%)	1 (0.5%)	1 (0.5%)					
	* 1/* 3B	2 (1.1%)								
	* 1/* 3C	1 (0.5%)								
Zabala-Fernández et al. (2011) [13]	* 1/* 1	217 (93.5%)	9 (3.9%)	5 (2.2%)	15 (6.5%)	14 (5.6%)	18 (7.8%)			
	* 1/* 2	3 (1.3%)	1 (0.4%)							
	* 1/* 3A	11 (4.7%)	2 (0.9%)		1 (0.4%)		1 (0.4%)			
	* 1/* 3C	1 (0.4%)								
Zhu et al. (2016) [42]	* 1/* 1	245 (96.8%)						62 (24.5%)		
	* 1/* 3C	8 (3.2%)						3 (1.2%)		

Abbreviations: TPMT: Thiopurine S-methyl-transferase enzyme.

**Table 5:** Association between TPMT polymorphisms and adverse effects.

Authors (Year)	TPMT Genotypes	Patients with AEs	Patients without AEs		(IC 95%)	*p*-Value
AL-Judaibi et al. (2016) [22]	variant (* 3A)	3 (17.6%)	2 (5.6%)	OR = 3.64	0.55–24.23	0.3127
	wild (* 1/* 1)	14 (82.4%)	34 (94.4%)			
Ansari et al. (2008) [23]	variants	15 (79.0%)				
	* 1/* 1	66 (35.0%)				
Coelho et al. (2016) [1]	TPMT * 3A	5 (55.6%)	4 (44.4%)			
	* 1/* 1	9 (13.1%)	60 (86.9%)			
Coenen et al. (2015) [6]	variants	9 (12.2%)		RR = 0.11	0.01–0.85	
	* 1/* 1	51 (7.3%)		RR = 1.2	0.72–2.09	
Coucoutsi et al. (2017) [11]	variants	3 (3.3%)	14 (15.4%)	OR = 8.87	0.97–81.11	0.048
	* 1/* 1	20 (22.0%)	54 (59.3%)			
Gazouli et al. (2010) [3]	* 1/* 1	1 (1.0%)	85 (87.6%)			
	variants	4 (4.1%)	7 (7.2%)			
Hlavaty et al. (2013) [31]	* 1/* 1	62 (30.2%)	143 (69.8%)	OR = 15.0	3.3–68.5	0.00002
	variants	13 (86.7%)	2 (13.3%)			
Ribaldone et al. (2019) [17]	* 1/* 1	58 (29.0%)	134 (67.0%)	OR = 0.77	0.08–7.7	0.82
	variants	2 (1.0%)	6 (3.0%)			
Wroblova et al. (2012) [9]	* 1/* 1	34 (18.1%)	138 (73.4%)			
	variants	10 (5.3%)	6 (3.2%)		2.124–17.094	<0.01
Zelinkova et al. (2006) [40]	* 1/* 1	3 (1.1%)	235 (89.8%)	OR = 6.316	2.141–18.634	0.004
	variants	4 (1.5%)	20 (7.6%)			
Zhu et al. (2016) [42] ^a^	* 1/* 1	62 (24.5%)	183 (72.3%)	OR = 1.21	0.74–1.97	0.44
	* 1/* 3C	3 (1.2%)	5 (2.0%)			

Note: OR: Odds ratio; RR: relative risk; “^a^” authors only evaluated the adverse reaction leukopenia.

**Table 6:** Association between clinical response and TPMT polymorphisms.

Authors (Year)	TPMT Genotypes	Response			OR	CI 95%	*p*-Value
		Reduced	Expected	Indeterminate			
AL-Judaibi et al. (2016) [22]	variants	2 (7.7%)	2 (12.5%)		0.583	0.074–4.615	0.628
	* 1/* 1	24 (92.3%)	14 (87.5%)				
Ansari et al. (2008) [23]	variants	-	55 (81.0%)				<0.001
	* 1/* 1	-	24 (43.0%)				
Coelho et al. (2016) [1]	variants	10 (76.9%)	22 (43.1%)				0.003
	* 1/* 1	3 (23.1%)	28 (54.9%)				
Dubinsky et al. (2000) [26]	variants	-	8 (8.7%)				
	* 1/* 1	15 (16.3%)	39 (42.4%)				
Gazouli et al. (2010) [3]	variants	-	11 (11.3%)				
	* 1/* 1	3 (3.1%)	83 (85.6%)				
Lee et al. (2017) [5]	variants	45 (32.1%)	90 (64.3%)				<0.001
	* 1/* 1	-	5 (3.6%)				
Palmieri et al. (2007) [15]	variants	45 (10.7%)	45 (10.7%)				
	* 1/* 1	45 (10.7%)	304 (72.0%)				
Stocco et al. (2005) [8]	variants	2 (40.0%)	3 (60.0%)				
	* 1/* 1	17 (33.3%)	34 (66.7%)				
Zabala-Fernández et al. (2011) [13]	variants	8 (3.5%)	4 (1.7%)	3 (1.3%)	2.74	0.81–9.22	0.096
	* 1/* 1	125 (53.9%)	30 (12.9%)	62 (26.7%)			

Note: OR: Odds ratio; CI: confidence interval.

## Data Availability

The datasets used in this study can be found in the full-text, tables and references that were included in the systematic review and are available on request from the corresponding authors.
